# Characterisation of the long-term effects of anthracycline-associated myocardial toxicity using cardiac MRI; a pilot study

**DOI:** 10.1186/1532-429X-11-S1-P95

**Published:** 2009-01-28

**Authors:** Marina L Hughes, Frederique Bailliard, Andrew Taylor, Gill Levitt

**Affiliations:** 1 Great Ormond Street Hospital for Children, London, UK; 2 North Carolina Children's Hospital, Chapel Hill, NC USA

**Keywords:** Anthracycline, Childhood Cancer, Anthracycline Dose, Myocardial Toxicity, Gadolinium Imaging

Cardiac MR (CMR) is the imaging modality with the best potential to characterise the myocardial effects of anthracycline therapy in the short and long term. We sought to assess this potential in adult long-term survivors of childhood cancer. Subjects were identified from our hospital's Oncology database, and were specifically selected if they had had acute leukaemia or Wilms tumour, had received anthracycline treatment for childhood cancer without radiotherapy more than ten years prior, and had suffered cardiotoxicity at any time, recognised by an echocardiographic fractional shortening <25%.

Of a total of 43 patients fitting these criteria, 18 (11 female) were contactable and all agreed to be scanned. Their median (range) age at cancer diagnosis was 4 (1 – 12) years, the total anthracycline dose ranged from 180 – 360 g/m^2^, and the median time since cessation of chemotherapy was 16 (11 – 26) years. All patients scanned claimed to be asymptomatic.

Four male patients had normal LV volumes and systolic function. The remaining 14 patients all had elevated LV end-systolic volumes, and 5 of these had elevated LV end-diastolic volumes indexed for body size. The median (range) LV ejection fraction (LVEF) was 55% (33–71%), with LVEF < 50% in 3/17 (all female) patients. The indexed LV mass was low for the whole group, with median (range) mass being 62 (54 – 79) g/m^2^ for males and 53 (43 – 71) g/m^2^ for females. Following gadolinium, and using conventional techniques, there was no evidence of late enhancement of the myocardium.

**Figure 1:**
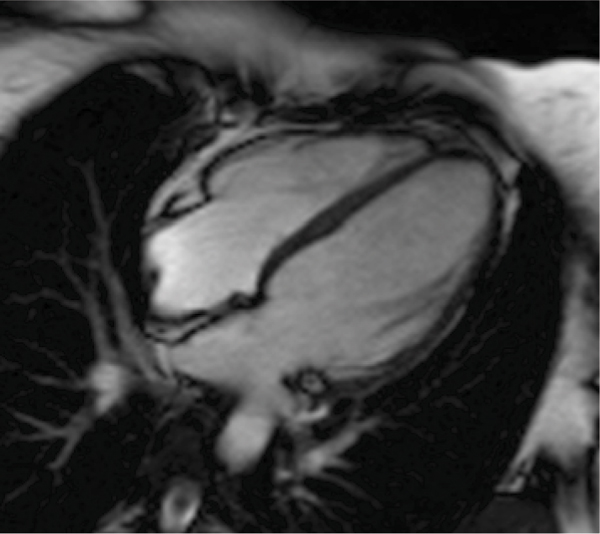
Figure 1

**Figure 2:**
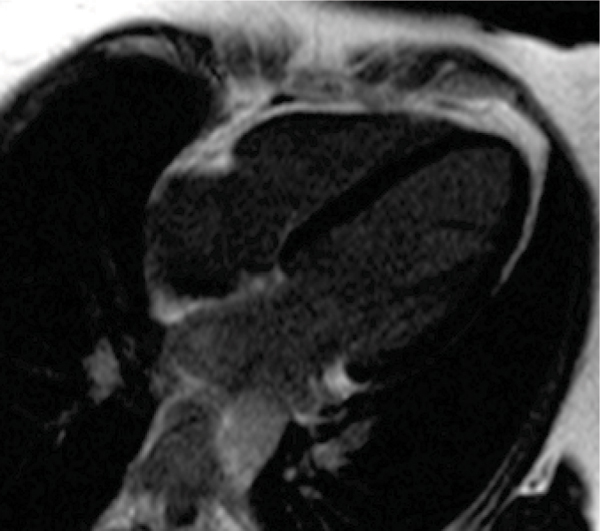
Figure 2

This pilot study specifically assessed patients identified on echocardiography to have had anthracycline-induced cardiac damage. A minority of these patients were found to have normal CMR findings. Most patients had poorly contractile LV myocardium, with low LV mass. Fibrosis or scarring, sought using conventional late gadolinium imaging techniques, was not evident in any patient. Further work is needed to find discriminative CMR indices to describe the long-term effects of myocardial toxicity and to predict prognosis in this patient group. See Figures 1 and 2.

